# Thought contents during rest account for functional connectivity-behavior associations

**DOI:** 10.1162/IMAG.a.55

**Published:** 2025-06-20

**Authors:** Junhong Yu, Yi-Sheng Wong, Charly Hugo Alexandre Billaud

**Affiliations:** Psychology, School of Social Sciences, Nanyang Technological University, Singapore; Division of Mathematical Sciences, School of Physical & Mathematical Sciences, Nanyang Technological University, Singapore

**Keywords:** resting-state, functional connectivity, behavior, mind wandering, spontaneous cognition

## Abstract

Resting-state functional connectivity (rsFC) is widely studied in relation to behavioral phenotypes. Typically, rsFC-behavior relationships are explained by attributing various psychological processes to the observed patterns of rsFC. Such explanations may seem far-fetched and unrelatable since subjects do not perform tasks that elicit these psychological processes during rest. Instead, they engage mostly in mind wandering. During such experiences, behavioral phenotypes can account for one’s resting-state thought contents (rsTC), which, in turn, explain variability in rsFC. This study posits that rsTC can explain the relationship between rsFC and diverse behavioral phenotypes. We analyzed 765 resting-state fMRI runs from 164 subjects (each completed at least 3 runs) in relation to self-reported rsTC ratings and 63 behavioral phenotypic scores; rsTC ratings were collected using the Short Version of the New York Cognition Questionnaire after each run. In a series of linear mixed-effect analyses on the within-subject data, we showed that similarity in rsTC across runs, especially vigilance-related TC, significantly predicted similarity in rsFC fingerprints, and several dimensions of rsTC can be significantly predicted by one or more network-to-network connections. Cross-sectionally, canonical correlation analysis with behavior as the outcome was used to derive canonical variates from the multivariate rsFC and rsTC predictors. For 45 of the behavioral phenotypes examined, their rsTC canonical variates significantly mediated their respective rsFC canonical variate-behavior associations. Overall, we have rigorously shown that rsTC is significantly related to rsFC and behavior, and importantly, rsTC can partially account for most rsFC-behavior relationships examined in this study.

## 1 Introduction

Owing to the relative ease of conceptualizing and executing a resting-state functional magnetic resonance imaging (rsfMRI) study, rsfMRI studies have been widely carried out, especially in relation to studying behavioral phenotypes. In these studies, the rsfMRI data are often analyzed in terms of functional connectivity (FC). With the advent of FC-based machine-learning approaches which are designed to produce generalizable behavioral predictions across datasets (Shen et al., 2017), resting-state functional connectivity (rsFC) investigations on behavioral phenotypes have proliferated to an even greater extent. In general, these rsFC studies have informed us a great deal about the diverse range of brain-behavior associations. Furthermore, these brain-behavior associations also possess some translational value; they can be used to predict behaviors or used as biomarkers for various clinical syndromes.

Unlike in a task-based fMRI study where various psychological processes relating to the hypothesis are manipulated, it is highly unlikely that psychological processes (e.g., executive control) relevant to the behavior of interest are elicited during the resting-state scan in a typical rsFC-behavior study. Despite this, it is fairly common to interpret these rsFC-behavior associations by drawing links to the widely assumed functions of various resting-state networks. For instance, the frontoparietal network (FPN) is widely thought to be involved in higher-order executive control owing to evidence from task-based fMRI studies (Rodríguez-Nieto et al., 2022). For this reason, it would be tempting to interpret the disrupted connectivity to or within the FPN in the context of a patient group as neurobiological evidence indicative of impaired executive processing—even though the resting-state ‘task’ does not require the subject to engage in any executive control processing at all. Such interpretations are analogous to inferring that a smaller hippocampal volume is suggestive of impaired learning and memory; of note, this is not true all the time (Molnár & Kéri, 2014). Therefore, in the above-mentioned frontoparietal connectivity example, rsFC interpretations have been reduced to a structure-function relationship, without consideration for the subject’s state of mind during the scan.

Given the issues discussed above, attempts at explaining the relationship between rsFC and behavior can seem rather abstract and far-fetched. However, this need not be the case; we can measure the thought contents during the resting-state scan, and use such thought contents to bridge the relationship between rsFC and behavior (Gonzalez-Castillo et al., 2021). On one end of this bridge, variation in resting-state thought contents (rsTC) can be linked to rsFC patterns. For instance, the variations in spontaneous past and future thoughts experienced during rest were found to be associated with rsFC between the medial temporal lobe and the default mode network (Andrews-Hanna et al., 2010). On the other end of the bridge, certain behavioral phenotypes may be linked to the increased preponderance of certain thought contents during resting-state. For instance, during mind-wandering episodes and resting-state fMRI scans, depressed patients experienced more negative, less positive, and more self- and past-related thoughts compared to healthy controls (Hoffmann et al., 2016;Li et al., 2024).

The current study explored this bridging explanation in detail. First, using within-subject measurements of rsFC and rsTC, we comprehensively investigated the relationship between rsTC and rsFC at the network-to-network connectivity and whole-brain FC fingerprint (i.e., whole-brain region-of-interest to region-of-interest FC profiles) levels. While there have been between-subject (Andrews-Hanna et al., 2010;Karapanagiotidis et al., 2017;Tarailis et al., 2024;Vatansever et al., 2020) and within-subject (Brennan et al., 2021;Stoffers et al., 2015) investigations on the rsFC correlates of rsTC, such research was limited by the fact that the dimensions of rsTC were analyzed in a univariate manner. For instance, in one study the dimension of ‘sleepiness’ was investigated in isolation from other thoughts and feelings during the resting-state scan (Stoffers et al., 2015), despite the fact that these other thoughts and feelings may co-occur with the feeling of sleepiness. Given that these other thoughts and feelings may contribute to differing patterns of rsFC, the exclusion of these variables will potentially confound the relationship between rsTC and rsFC. In the current within-subject analyses, in addition to mapping out the network-to-network connections associated with each rsTC dimension in a univariate manner, we also examined how similarity across multiple rsTC dimensions is related to similarity in whole-brain rsFC fingerprint. This will provide a more conclusive and comprehensive estimate of the strength of the relationship between rsFC and rsTC in general.

Second, we examined how well rsTC can significantly predict a diverse range of phenotypes. While previous research has examined rsTC in relation to a certain behavioral phenotype of interest (e.g., depression (Hoffmann et al., 2016)), we aimed to scale this up by examining the associations between rsTC and a diverse array of behavioral outcomes included in a richly behaviorally-phenotyped dataset (Mendes et al., 2019). Upon establishing that behavioral phenotypes are significantly associated with both rsTC and rsFC, we then proceeded to examine whether rsTC can mediate the relationship between rsFC and behavior in order to validate our proposed ‘bridging’ explanation. The aims of the current study can be summarized with the following hypotheses:

1) Similarity in rsTC significantly predicts similarity in whole-brain rsFC fingerprints
2) Each of the rsTC dimensions can be significantly predicted by one or more network-to-network connections
3) rsTC significantly mediates the relationship between rsFC and behavior

## 2 Methods

### 2.1 Participants and procedures

The present study analyzed data from the MPI-Leipzig Mind-Brain-Body database (Mendes et al., 2019). The MPI-Leipzig Mind-Brain-Body study recruited participants who did not meet any of the following exclusion criteria: 1) history of psychiatric, neurological, and malignant disorders; 2) currently consuming centrally active drugs, beta- and alpha-blockers, cortisol, or any chemotherapy or psychopharmacological medications; 3) had extensive testing experience at the Max Planck Institute for Human Cognitive and Brain Sciences or any other academic institutions; 4) is a current or previous student of psychology; and 5) had any MRI contraindications. The database’s study protocol was approved by the ethics committee at the medical faculty of the University of Leipzig (097/15-ff). For each subject, data collection took place across 5 days; the MRI scans were acquired on day 3.

This database included 194 native German-speaking participants. Among them, we analyzed 164 participants who had at least 3 out of 4 valid (i.e., without excessive head motion) resting-state runs and matching valid data from the Short Version of the New York Cognition Questionnaire (SNYCQ) for each of these runs. Eleven of the analyzed subjects only had 3 valid runs of data (resting-state fMRI scans with matching SNYCQ data), while the rest had complete data for all 4 runs. In order to prevent identification of the original subjects, the exact ages were not provided by the authors of the database; instead, the ages of the participants were given in 5-year age bins. The distributions of these age bins and sex among the analyzed subjects are shown inFigure 1a.

**Fig. 1.**
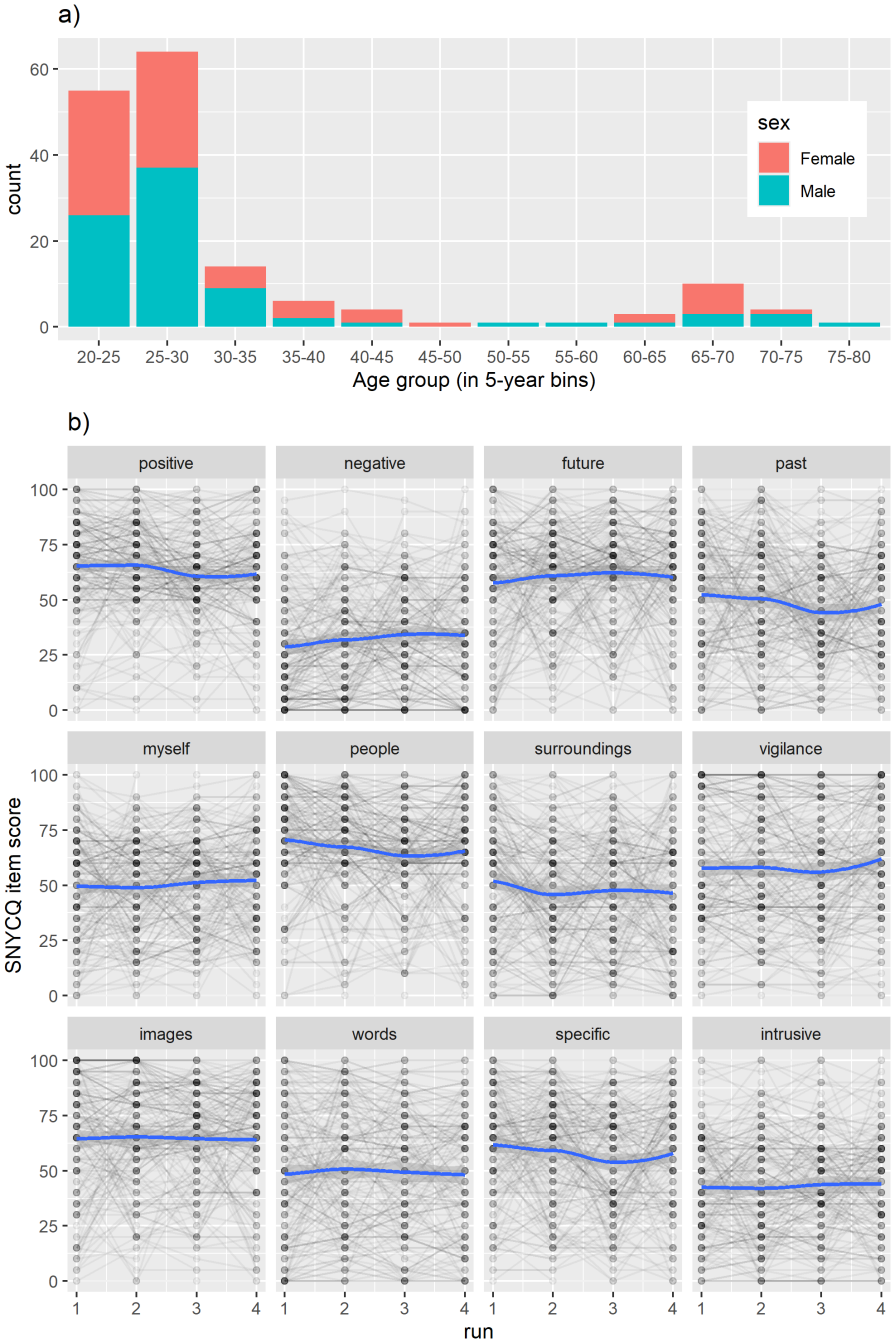
(a) Age- and sex-distributions of the analyzed sample. (b) Subject-level (gray lines) and group-averaged (thick blue lines) trajectories for each of the 12 SNYCQ items. The group-averaged trajectories were estimated using the locally estimated scatterplot smoothing (LOESS) procedure as implemented in the ‘ggplot2’ R package.

### 2.2 Resting-state thought contents

The SNYCQ (Gorgolewski et al., 2015) was administered after each resting-state scanning run. This questionnaire was adapted from the New York Cognition Questionnaire (Gorgolewski et al., 2014) and included 12 items to assess the content and form of self-generated thoughts during the scan. Participants rated each of these items on a sliding scale ranging from 0 (‘Completely did not describe my experience’) to 100 (‘Completely described my experience’) in increments of 5. The SNYCQ item scores, across all four runs, are illustrated inFigure 1b.

### 2.3 MRI acquisition

Participants were scanned using a 3T scanner (Magnetom Verio, Siemens Healthcare, Erlangen, Germany) equipped with a 32-channel Siemens head coil at the Day Clinic for Cognitive Neurology, University of Leipzig. Structural images were acquired using a 3D MP2RAGE sequence (TE = 2.92 ms; TR = 5000 ms; TI1 = 700 ms; TI2 = 2500 ms; FOV = 256 x 240 x 176 mm; voxel size = 1 mm isotropic). Each participant completed four runs of resting-state fMRI scans in a single session. During the resting-state fMRI scan, subjects were instructed to remain awake and lie still with their eyes open while looking at a fixation cross. In each run, 657 T2*-weighted gradient-echo EPI volumes were acquired (voxel size = 2.3 mm isotropic, FOV = 202 × 202 mm^2^, imaging matrix = 88 × 88, 64 slices, TR = 1400 ms, TE = 39.4 ms, flip angle = 69°, echo spacing = 0.67 ms). The first and third runs were acquired in the anterior-posterior phase encoding direction, while the second and fourth runs were acquired in the posterior-anterior phase encoding direction. Additionally, a pair of spin echo images were acquired to correct for distortions in the fMRI scans (voxel size = 2.3 mm isotropic, FOV = 202 × 202 mm^2^, imaging matrix = 88 × 88, 64 slices, TR = 2200 ms, TE = 52 ms, flip angle = 90°, echo spacing = 0.67 ms).

### 2.4 MRI preprocessing

The fMRI scans were preprocessed using fMRIPrep 24.0.1 (Esteban et al., 2019). Functional data were slice-time corrected using 3dTshift from AFNI (Cox, 1996) and motion-corrected using MCFLIRT (Jenkinson et al., 2002). This process was followed by co-registration to the corresponding T1w using boundary-based registration (Greve & Fischl, 2009) with 9 degrees of freedom, using bbregister from FreeSurfer (Fischl, 2012). Motion correcting transformations, BOLD-to-T1w transformation, and T1w-to-template (MNI) warp were concatenated and applied in a single step using antsApplyTransforms employing Lanczos interpolation. Subsequently, these preprocessed volumes were denoised by regressing out 6 motion parameters, the average signal of white matter and cerebrospinal fluid masks, global signal, and their derivatives, as well as cosines covering slow time drift frequency band using the ‘nilearn’ library in Python. The volumes were then subjected to a 0.1 Hz low-pass filter. Runs with excessive head motion, as defined by having a mean root mean squared displacement greater than 0.25 mm, are excluded from the analyses.

The preprocessed timeseries data were parcellated into 200 cortical nodes using the Schaefer-200 atlas (Schaefer et al., 2018) and 19 subcortical nodes using the FreeSurfer subcortical segmentations (Fischl et al., 2002). Then, for each subject, a 219 x 219 FC matrix was generated from the bivariate Fisher-Z transformed correlations between nodes in the parcellated timeseries. We vectorized all FC matrices by transforming the bottom triangle of the FC matrix into a single column of values (i.e., FC vectors) prior to the analyses.

### 2.5 Statistical analyses

First, we examined whether similarity in SNYCQ scores was related to similarity in FC fingerprints at the within-subject level. For each subject, we calculated the mean absolute differences (MADs) in the FC vectors and 12-item SNYCQ scores between every unique pair of runs. For instance, the MAD for the FC vectors between runs 1 and 2 was calculated by first computing the edge-wise absolute differences between both runs, and then taking the mean of these edge-wise absolute differences. The MAD for all the SNYCQ scores (full-scale level) was calculated in a similar manner. For a subject with 4 valid runs, there would be 6 pairs (FC vectors and SNYCQ scores) to calculate MADs from. The data were then fitted to a linear mixed-effects model. Specifically, the MADs for the FC vector and SNYCQ scores were designated as the outcome and predictor, respectively, and a random intercept was modeled for each subject. These steps are summarized inFigure 2. Subsequently, to examine how similarity in specific rsTC dimensions was related to the similarity of rsFC fingerprints, we repeated this analysis by using the absolute difference of each SNYCQ item (as opposed to the MAD of 12-item SNYCQ scores), individually, as the predictor in the linear mixed-effects model.

**Fig. 2.**
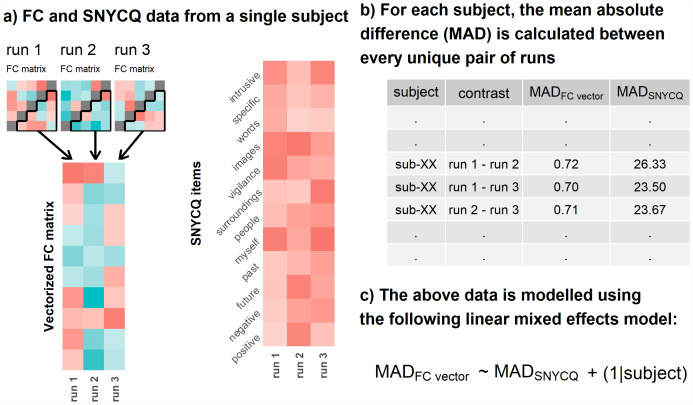
Overview of the FC fingerprinting analyses. (a) A hypothetical example of the data analyzed for each subject. We use an example of a subject with 3 runs of data for simplicity and brevity’s sake even though most participants had 4 valid runs of data. Likewise, a 5 x 5 FC matrix, instead of the 219 x 219 FC matrix, was illustrated for simplicity. (b) The mean absolute differences (MAD) between each pair of unique runs were calculated for the FC vectors and SNYCQ scores. These MADs were tabulated in a long format to facilitate the subsequent linear mixed-effects analysis. (c) The data are fitted to a linear mixed-effects model, where the MADs for the FC vector and SNYCQ scores were the dependent and independent variables, respectively, and a random intercept is fitted for each subject.

Next, to determine the resting-state network correlates for each of the SNYCQ items, we first summarize the 219 x 219 FC matrix into an 8 x 8 functional network connectivity (FNC) matrix (including the 7-networks parcellation plus the network of subcortical regions). Briefly, we grouped the edges from the FC matrix into their respective network-to-network groupings (Schaefer et al., 2018) and computed the average edge value within these groups. Unlike the 219 x 219 FC matrix, the values along the diagonal in the 8 x 8 network FNC matrix were computed and analyzed as well. These values represent the within-network connectivity. The FNC matrix contains 64 edges; among them, 36 were between unique pairs of networks. Then, we fitted another array of linear mixed-effect models with the edges in the FNC matrix as the outcome, the SNYCQ item as the predictor, and a random intercept for each subject. Altogether 432 (36 unique FNC edges x 12 SNYCQ items) models were fitted in these analyses. For each SNYCQ item predictor separately, we applied false discovery rate (FDR) correction to correct for its corresponding 36 tests, instead of correcting for 432 tests collapsed across all sets. These linear mixed-effect analyses were carried out using the ‘lmerTest’ R package. The standardized coefficient of the predictor and its 95% confidence intervals were estimated using the ‘standardize_parameters()’ function from the ‘effectsize’ R package.

Prior to the next set of analyses, the data were prepared in a different manner. In the original dataset, each subject had up to 4 runs of SNYCQ and FC data. In the interest of parsimony, instead of including up to 4 x 12 SNYCQ items as predictors in the subsequent analysis, we computed the across-run average for each of the 12 SNYCQ items and these averaged values were used in the subsequent analyses. We did not average the FNC data in a similar manner. Instead, the preprocessed timeseries data were concatenated across runs for each subject and new 219 x 219 FC matrices were computed from these concatenated timeseries data, which were subsequently summarized into 8 x 8 FNC matrices.

Canonical correlation analysis (CCA), as implemented in the R package ‘yacca’ (Butts, 2022), was used to examine the multivariate associations between SNYCQ items, FNC edges, and behavioral phenotypes. Briefly, given two sets of variables, CCA identifies linear combinations of the variables within each set that maximizes the correlation between sets. CCA has been used to relate multivariate neuroimaging features to single or multiple behavioral phenotypes in several studies (Zhuang et al., 2020). In the current analyses, we included in our analyses all behavioral phenotypes (e.g., total and sub-scale scores of various questionnaires) contained in the MPI-Leipzig Mind-Brain-Body database if at least 90% of the 164 analyzed subjects had valid data for the phenotypical variable. Essentially, we are using the CCA as a dimensional reduction technique to derive univariate rsTC and FNC summary scores (i.e., canonical variates [CV]), such that they can be entered into the subsequent mediation analyses. In the rsFC literature, the importance of a particular functional network varies depending on the behavior that is studied. For this reason, we do not want the same FNC summary/component scores to be used in analyses across all behavioral variables; hence, the use of principal component analysis would not be feasible. Instead, for each behavioral variable we used CCA to compute an FNC CV derived from multiple FNC edges, whose weights vary flexibly depending on the behavior in question. In a similar manner, we also computed an rsTC CV derived from the multiple SNYCQ items, whose weights vary flexibly depending on the behavior in question.

CCA was carried out twice for each behavioral phenotype. In the first CCA, the 12 SNYCQ items were entered as the first set of variables, and the behavioral phenotypic scores (as a single variable) were entered as the second set. The second CCA was similar to the first except that its first set consisted of the 36 unique FNC edges. Then, two CVs scores (i.e., SNYCQ and FNC) were computed through a matrix multiplication of the SNYCQ item scores/FNC edges with their respective loadings. These CVs represent the summary scores derived from linear combinations of their constituent variables. The behavioral phenotypic scores were then correlated with these CVs. Finally, for each behavioral phenotype, we ran mediation analyses, as implemented in the R package ‘psych’ (Revelle, 2024), with the CV_FNC_as the predictor, behavioral scores as the outcome, and CV_SNYCQ_as the mediator. These mediation analyses were intended to estimate the indirect effect of CV_SNYCQ_in mediating the relationship between CV_FNC_and behavior. The statistical significance of the indirect effect was assessed via its 95% confidence intervals, which were computed using 5000 bootstrapped samples. Briefly, the regression coefficients of the mediation paths A and B are computed, and these coefficients are multiplied (i.e., A*B) to obtain the indirect effect for the bootstrapped sample. This procedure is repeated for each of the 5000 bootstrapped samples, thus generating 5000 bootstrapped indirect effects—which the 95% confidence intervals were estimated from.

In these set of analyses, FDR correction was applied separately, to the statistical significance of the correlations between the CVs and behavior, as well as the indirect effects.

Power analyses were carried out for these analyses via 1000 simulations on artificially generated data using conservative parameters. These power analyses are reported in greater detail in theSupplementary Materials. Briefly, we estimated that these analyses had statistical powers of at least 87.9%. Statistical significance was set at*p *< .05 for all analyses. All analyses were carried out in R 4.3.2. The R codes for the analyses and generating the figures, together with the preprocessed data analyzed in this study are available athttps://osf.io/5hkdg/.

## 3 Results

### 3.1 rsTC is significantly associated with rsFC at the FC fingerprint and FNC levels

To test the associations between SNYCQ (12-item and individual items) scores similarity and FC similarity at the within-subject level, we analyzed the within-subject MADs, computed from the SNYCQ scores and FC fingerprints, using linear mixed-effects models. The results are shown inFigure 3a. Overall, similarity in FC was significantly predicted by similarity in the 12-item SNYCQ scores. Additionally, the item-level analyses revealed that similarity in the item scores of ‘myself’, ‘people’, ‘surroundings’, ‘vigilance’, ‘images’, and ‘specific’ significantly predicted similarity in FC fingerprints. However, after applying FDR correction to these 12 item-level tests, similarity in the item scores of ‘images’ no longer predicted similarity in FC fingerprints significantly (*p*_FDR_= 0.063). Of note, in terms of standardized coefficients, ‘vigilance’ (β = 0.28) outperformed, quantitatively, the full-scale SNYCQ (β = 0.23) in predicting FC similarity. In general, these standardized coefficients were small in magnitude.

**Fig. 3.**
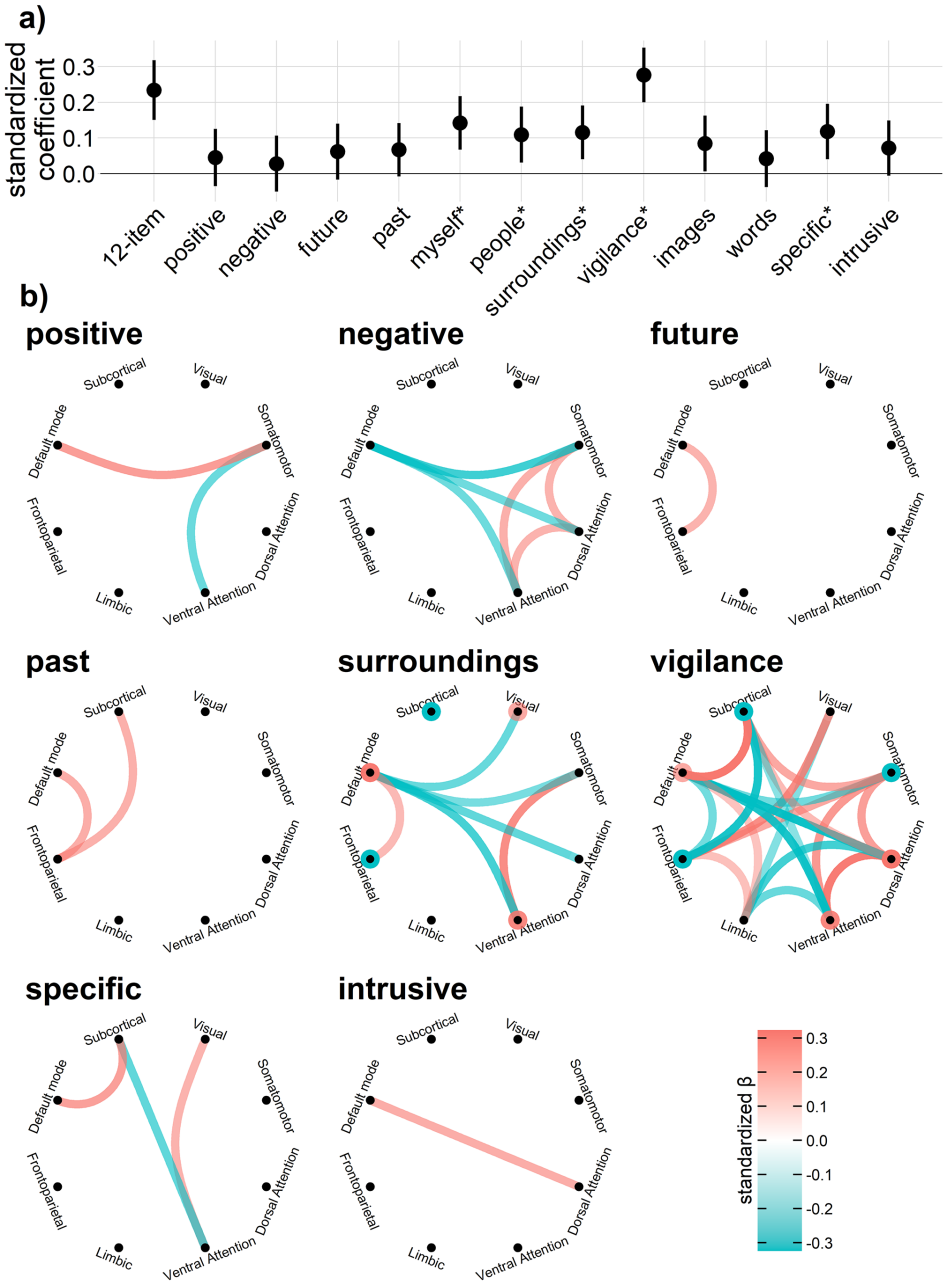
(a) Standardized coefficients and their respective 95% confidence intervals when the MAD_SNYCQ_and absolute differences in item scores were used to predict the MAD_FC_in linear mixed-effects models. These coefficients are statistically significant if their 95% confidence intervals (uncorrected for multiple comparisons) do not intersect with the y = 0 line. *Statistically significant after correcting for false discovery rate at the item level. (b) Connectograms for each of the SNYCQ items illustrating the standardized coefficient of the network-to-network connections that were significantly (*p*_FDR_< .05) predicted by the SNYCQ item scores. The rings around the nodes represent within-network connections. The SNYCQ items omitted from this plot did not yield any significant network-to-network connections.

There may be some concerns that the contrasts used in these analyses are overlapping, and hence the MADs are dependent on each other. For instance, the MADs for run 1 – run 2, and run 1 – run 3 may be more similar to each other, compared to run 1 – run 2, and run 3 – run 4; this dependency was not accounted for in the linear mixed-effects model. Hence, we have repeated these analyses, including only non-overlapping contrasts (e.g., run 1 – run 2 & run 3 – run 4). As presented in the Supplementary Materials, the results (seeFig. S1) from the analyses of these non-overlapping contrasts were similar to the results shown inFigure 3a.

Next, we examined the FNC correlates for each of the 12 SNYCQ dimensions at the within-subject level using linear mixed-effects models. After correcting for FDR, significant network-to-network connections emerged for 8 out of the 12 SNYCQ dimensions (seeFig. 3b). In particular, among the 12 SNYCQ items, ‘vigilance’ had significantly predicted the greatest number of network-to-network connections. The conditional and marginal R^2^(Nakagawa & Schielzeth, 2013) in these linear mixed-effects models are illustrated inFigures S2 and S3in the Supplementary Materials.

### 3.2 Canonical correlations between rsTC/rsFC and behavior

**Fig. 4.**
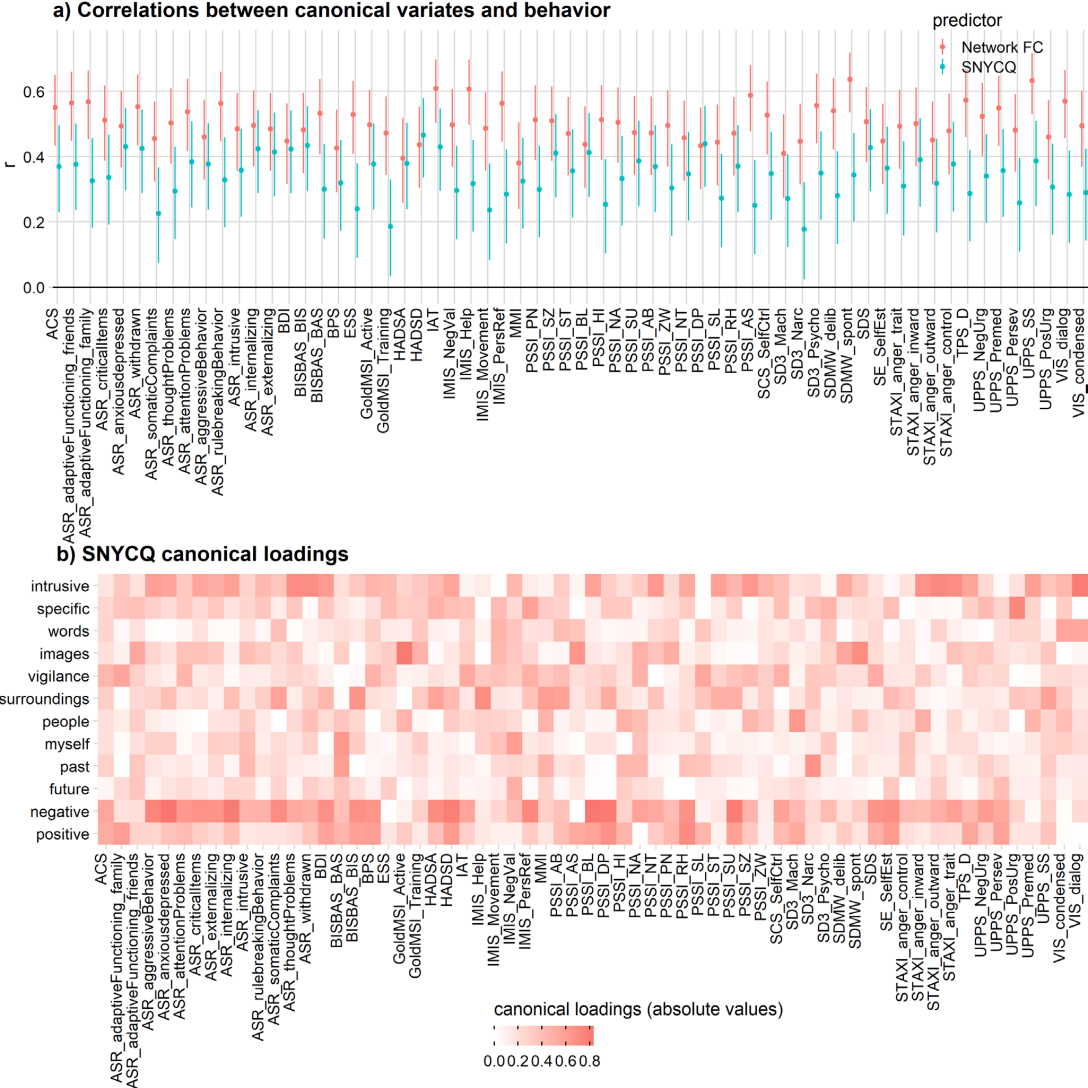
(a) Correlations between the CV_FNC_and CV_SNYCQ_and behavioral phenotypes. The error bars represent the coefficients’ 95% confidence intervals. These coefficients are statistically significant if their 95% confidence intervals do not intersect the y = 0 line. (b) Canonical loadings (absolute values) of SNYCQ items.

Two sets of CCAs were used to examine the multivariate associations between behavioral phenotypes and SNYCQ scores or FNC edges. A total of 63 behavioral phenotypical measures, which 90% of the current study’s sample had valid data for, were included in these analyses. The list of included measures is provided in the Supplementary Materials (seeTable S1). In these CCAs, both the CV_FNC_and CV_SNYCQ_were significantly correlated with all included behavioral phenotypes, even after applying FDR correction to 63 tests for each CV (seeFig. 4a).Figure 4billustrates the absolute canonical loadings for each SNYCQ item across all behavioral phenotypes; these loadings roughly indicate the importance of each SNYCQ item in relation to each of the behavioral phenotypes. The canonical loadings for the FNC edges are illustrated in a similar plot inFigure S4in the Supplementary Materials.

### 3.3 rsTC mediates the relationship between rsFC and most behavioral phenotypes

Next, mediation analyses were carried out for each behavioral phenotype, with CV_FNC_as the predictor, behavioral phenotypic score as the outcome, and CV_SNYCQ_as the mediator. As seen fromFigure 5b, the indirect effects derived from 47 of such analyses were statistically significant. Among the nonsignificant 16 indirect effects/mediation models, 12 of them had nonsignificant relationships between CV_FNC_and CV_SNYCQ_(i.e., path A), and 3 of them had nonsignificant CV_SNYCQ_-behavior relationships after controlling for CV_FNC_(i.e., path B) (seeFig. 5a). After applying FDR correction to the multiple indirect effect statistics, 45 of them remained statistically significant.

**Fig. 5.**
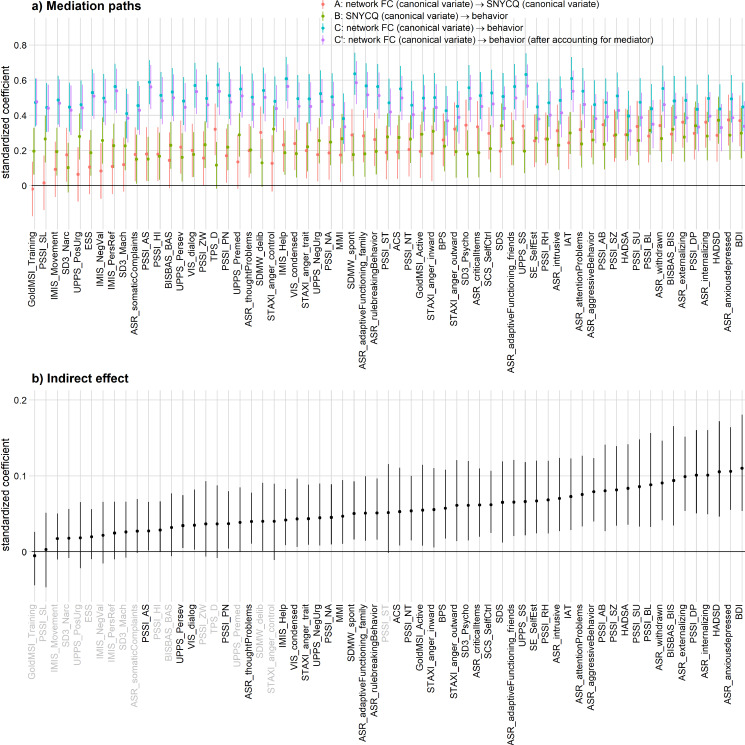
(a) Standardized coefficients of the three mediation paths (i.e., A, B, and C) and their confidence intervals. Path A represents the coefficient of CV_FNC_when CV_SNYCQ_is regressed on CV_FNC_. Path B represents the coefficient of CV_SNYCQ_when the behavioral outcome is regressed on CV_FNC_and CV_SNYCQ_. Path C represents the coefficient of CV_FNC_when the behavioral outcome is regressed on CV_FNC_. Path C’ represents the coefficient of CV_FNC_when the behavioral outcome is regressed on CV_FNC_and CV_SNYCQ_. (b) The indirect effect of CV_SNYCQ_in mediating the relationship between CV_FNC_and behavioral outcome and its confidence intervals. The error bars in both panels represent the coefficients’ 95% confidence intervals. These coefficients are statistically significant (uncorrected for multiple comparisons) if their 95% confidence intervals do not intersect the y = 0 line. The labels that are colored in gray correspond to indirect effects that were not statistically significant after correcting for false discovery rate. The full names of these measures and their references are provided in the Supplementary Materials (seeTable S1). For both panels, the behavioral phenotypes are arranged along the x-axis in order of increasing indirect effects.

**Fig. 6.**
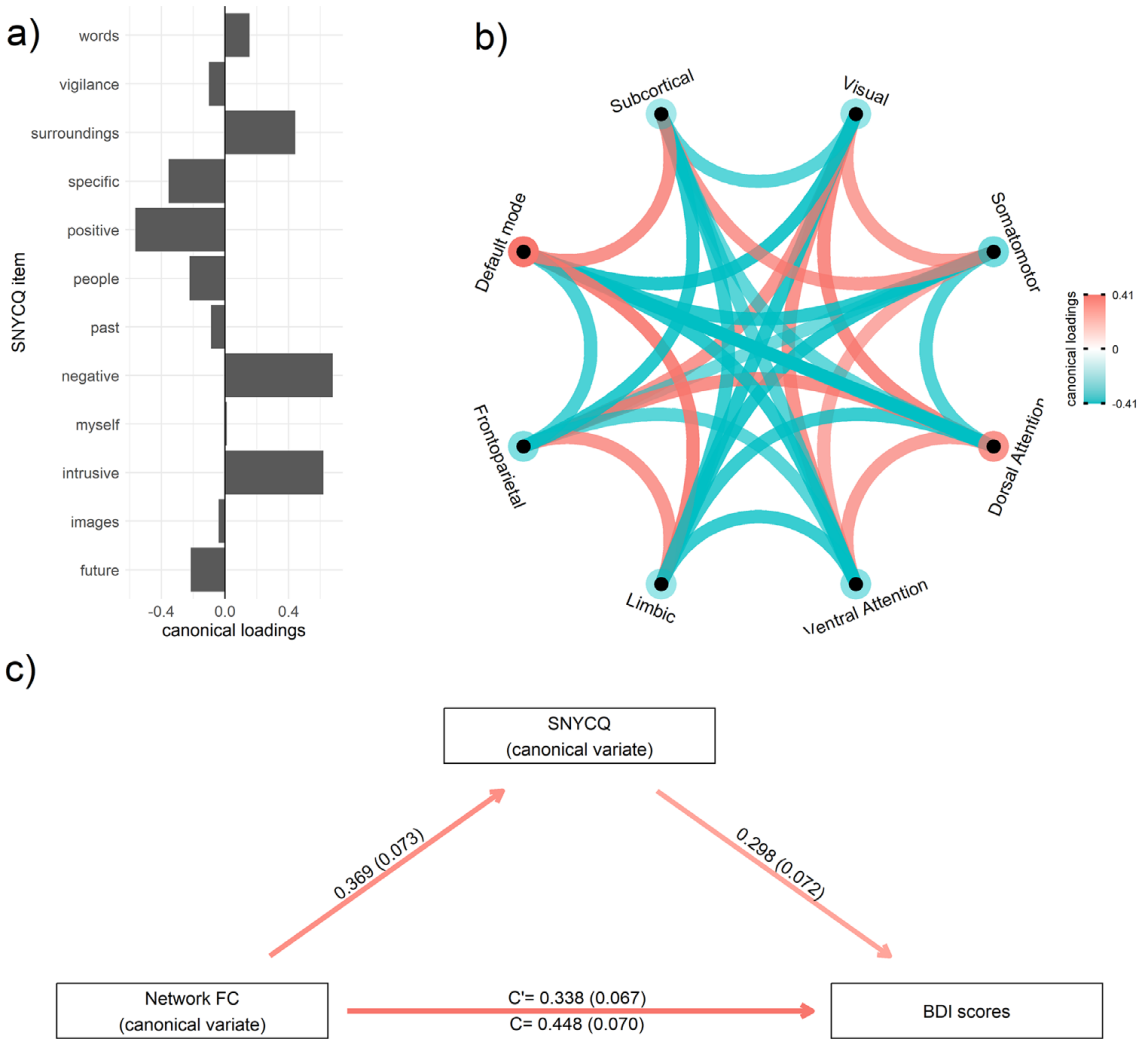
Example of the analyses involving the Beck Depression Inventory scores. (a) SNYCQ item loadings from the canonical correlation analysis and (b) network FC. (c) Mediation path diagram. All reported coefficients in the mediation path diagram are standardized and statistically significant (*p*< .001). The standard errors of the coefficients are provided in parentheses.

In order to better contextualize these findings, we present in greater detail (seeFig. 6), an example of these analyses involving a widely-studied behavioral phenotype—depression, as measured via the Beck Depression Inventory-II (Beck et al., 2011). In this example, one would notice that the canonical loadings were distributed in an expected manner across the SNYCQ items. For instance, the positive thoughts were loaded negatively and negative thoughts were loaded positively (Fig. 6a). Furthermore, the loadings on the FNC edges were somewhat consistent with these thought patterns as well. In the previous analyses (seeFig. 3b), a positive somatomotor-default mode and negative somatomotor-ventral attention network connections were associated with positive thoughts. In the context of depression, this connection was loaded in the reverse manner (seeFig. 6b); that is, negative loadings in the former and positive loadings in the latter. Additionally, the connections between the somatomotor, ventral attention, dorsal attention, and default mode networks were associated with depression (seeFig. 6b), these connections were loaded in a manner that was largely consistent FNC patterns associated with negative thoughts (seeFig. 3b). Overall, these findings showed how the pattern of loadings in the SNYCQ and FNC sets is somewhat consistent with the rsFC-rsTC associations we identified earlier.

In the mediation analyses, it was observed that the effect of CV_FNC_on BDI scores had dropped in magnitude (from 0.448 to 0.338) after accounting for the CV_SNYCQ_mediator. Nevertheless, this direct effect (i.e., C’) remained statistically significant (*p *< .001) which suggests that a partial mediation has occurred.

### 3.4 Supplementary analyses

Given the relatively wide age range of subjects included in these analyses, it is possible that age could exert some confounding effect on these results. To this end, we repeated all analyses while excluding 21 subjects who were older than 40. The results of these analyses are presented in the Supplementary Materials (pages 7–11;Figs. S5–S9). Generally, most of the findings hold in the 40-or-younger sample. Nevertheless, there were some salient differences. First, after correcting for FDR, similarity in the item scores of ‘intrusive’ significantly predicted similarity in FC fingerprints (seeFig. S5ain the Supplementary Materials); previously, this was not the case. Second, the analyses of within-subject FNC correlates no longer revealed significant FNC correlates for the SNYCQ dimensions of ‘intrusive’, ‘past’, and ‘future’ (seeFig. S5bin the Supplementary Materials). Finally, CV_SNYCQ_significantly mediated the relationship between CV_FNC_and behavior for 36 (45 in the original analyses) of the rsFC-behavior relationships (seeFig. S8in the Supplementary Materials).

## 4 Discussion

In the current study, we examined via a series of analyses whether the relationship between rsFC and behavior can be explained by rsTC. Our within-subject analyses showed that rsTC significantly predicted rsFC, both at the FC fingerprint and FNC levels. In particular, the relationship between rsTC and rsFC fingerprints appears to be mostly explained by the rsTC’s dimension of vigilance. Next, using CCA, we showed that different dimensions of rsTC can be combined linearly into CVs that were significantly associated with a vast array of behavioral phenotypes, and these rsTC CVs significantly mediated the majority (45 out of 63 after correcting for FDR) of the rsFC-behavior relationships examined in the current study. Overall, we have shown that rsTC is closely related to rsFC and more importantly, rsTC can meaningfully explain the relationship between rsFC and behavior.

As expected, we observed a significant association between similarity in rsTC and similarity in rsFC fingerprints, at the within-subject level. These results complement previous univariate findings on the relationship between rsTC and rsFC by showing that multiple rsTC characteristics contribute concurrently to variability in rsFC patterns. However, it should be noted that the overall relationship between rsFC and rsTC is small (standardized coefficient = 0.23). This small association may suggest that there could be several other rsTC characteristics unaccounted for, which could significantly account for variability in FC patterns. For instance, ‘discontinuity of mind’, and ‘comfort’ are two dimensions of rsTC assessed in the Amsterdam Resting-State Questionnaire (Diaz et al., 2013) that had no equivalents in the SNYCQ; ‘discontinuity of mind’ was found to be associated with FC alterations in the visual and somatomotor networks, while ‘comfort’ was associated with FC in the somatomotor network (Stoffers et al., 2015) and globally in the brain (Tarailis et al., 2024).

Furthermore, this weak relationship between rsTC and rsFC is also consistent with the notion that rsFC and rsfMRI signal variability are only minimally explained by ongoing cognition. For instance, the brain’s blood flow, measured via PET, fluctuates only around 5% across task and resting states (Raichle & Mintun, 2006); if cognition were a major component of rsfMRI signal variability, we would expect much larger fluctuations. Additionally, patterns of DMN connectivity that were observed during rest were also observed to be present when conscious thought was minimal or absent, such as during sleep (Larson-Prior et al., 2009) or light sedation (Greicius et al., 2008). rsfMRI signal variability was also largely influenced by the neuronal cellular metabolic state and aerobic glycolysis levels (Raichle, 2015). Non-neural factors such as respiratory and cardiac activity, and head motion can also contribute significantly to variability in rsFC (Fox & Raichle, 2007;Laumann et al., 2024). While the consequences of head motion on rsFC have been largely mitigated as a result of our fMRI denoising procedures, the influences of respiratory and cardiac activity on rsFC remain largely adjusted for in the current study. In particular, it was shown that measures of respiratory activity, such as the envelope of the waveform, windowed variance in the waveform, and respiration volume per time, varied systematically across time, during resting-state fMRI scans in the Human Connectome Project Young Adult Study (Power et al., 2020). Thus, it is very likely that such respiratory-related noise could potentially weaken and obscure the relationship between rsFC and rsTC in our analyses.

As we analyzed the relationship between similarity in FC fingerprints and similarity in single-item SNYCQ scores, it became clear that the rsTC-rsFC fingerprint relationship was largely explained by variability in the ‘vigilance’ rsTC dimension. In the current study, vigilance was measured with the item ‘I was fully awake’. A previous review (Liu & Falahpour, 2020) has documented widespread alterations in FC as a result of vigilance-related changes attributable to sleep deprivation (Yeo et al., 2015), drugs (Licata et al., 2013), and caffeine intake (Wong et al., 2012). Our results further add to these findings in showing that even changes in vigilance across an hour of resting-state scans can significantly influence FC patterns; importantly, these changes in FC patterns occur in the absence of the psychopharmacological confounds typically associated with the intake of caffeine and drugs.

In our univariate analyses on the FNC correlates of various rsTC dimensions, we obtained results that were largely different from those of previous studies (Andrews-Hanna et al., 2010;Brennan et al., 2021;Karapanagiotidis et al., 2017). That being said, it is difficult to make a direct comparison between our findings with those of previous research for various reasons. First, as alluded to previously, different questionnaires were used in previous studies to characterize the different dimensions of rsTC, and some of these do not have equivalents in the SNYCQ. As for research that studied dimensions of rsTC similar to those examined in the SNYCQ, they had largely analyzed FC in a different manner. We have uncovered rsFC correlates of ‘positive’, ‘negative’, ‘surroundings’, and ‘specific’ where previous studies (Brennan et al., 2021;Karapanagiotidis et al., 2017) have largely failed. Furthermore, two cross-sectional studies have linked mental time traveling (thoughts about past and future) to DMN connectivity with the medial temporal lobe (including the hippocampus); in relation to these findings, our results revealed that thoughts about the past and future were significantly associated with connectivity between the default mode and frontoparietal networks. The latter network is nowhere near the medial temporal lobe region. Notwithstanding differences in MRI acquisition and preprocessing, these studies (Andrews-Hanna et al., 2010;Brennan et al., 2021;Karapanagiotidis et al., 2017) had analyzed FC at much finer scales (e.g., relatively small ROI seeds or ROI-to-ROI connectivity), and thus our analyses on the rsFC, averaged across large networks, may not be sensitive enough to detect certain associations that are likely to be mapped to anatomically specific regions in the brain.

It is also worth pointing out that a previous study (Hardikar et al., 2024) had used the same dataset to study a similar topic. It derived an ‘internal specific thought’ composite score via principal component analyses on the 12 SNYCQ items; the SNYCQ items of ‘specific’ and ‘vigilance’ loaded heavily on this composite score. The authors showed that the gradient scores of the extrastriate cortex and bilateral superior parietal lobule from the first 3 FC gradients were significantly associated with this ‘internal specific thought’. While we did observe that connections to the visual network, within which the extrastriate cortex is located, were significantly associated with ‘vigilance’ and ‘specific’, our methods were too different to infer commonalities between our results and theirs meaningfully.

Our CCA analyses showed that rsTC multivariate features were significantly associated with a diverse array of behavioral phenotypes. As expected, the SNYCQ ‘negative’ item loaded heavily on behavioral phenotypes in the affective domains. Overall, we showed that the rsTC multivariate features significantly mediated several rsFC-behavior relationships. A previous study (Vatansever et al., 2020) reported that thought patterns, specifically varying in importance and specificity, significantly mediated the relationship between rsFC and psychological well-being. We were able to replicate this mediation effect with well-being-related dependent variables (e.g., depression, anxiety, and psychiatric symptom scores), and many other dependent outcomes unrelated to well-being (e.g., personality) as well. In cases where the rsTC multivariate features did not significantly mediate the rsFC-behavior associations, this occurred largely because the multivariate rsTC and rsFC features did not relate well to each other. In these cases, the FNC patterns associated with the behavioral outcomes may possibly encode other aspects of rsTC that were not captured by the SNYCQ.

It should be pointed out that the use of the rsTC CVs in these mediation analyses brings about certain nuances to the interpretation of these results. It meant that not all of the rsTC dimensions were equally important in mediating the rsFC-behavior relationship, and the importance of each of these dimensions varies across different behavioral phenotypes. As we have shown in the BDI example, negative thoughts loaded heavily on the rsTC CV; however, in the example of Epworth Sleepiness Scale (ESS; seeFig. 4b), negative thoughts were nowhere near as important. In this regard, future research in this area may want to examine specific dimensions of rsTC that are hypothesized to mediate rsFC-behavior associations.

These findings are subjected to various limitations. First, rsTC was measured subjectively via a retrospective recall. As such, the accuracy of such self-reported rsTC, can be influenced by one’s recall ability, as well as primacy and recent biases (Stawarczyk & D’Argembeau, 2019)—thought contents that occurred in the middle of the scan were less likely to be recalled compared to those toward the start and end of the resting-state scan. Second, the use of SNYCQ or any questionnaire to assess rsTC grossly simplifies the mind-wandering experience. As alluded to repeatedly in this report, the 12 SNYCQ items cannot possibly capture all behaviorally relevant information during our mind-wandering experience. Furthermore, such rsTC measurements also ignore the dynamics of mind wandering—how thought contents change over time (Christoff et al., 2016). Such dynamics could potentially encode behaviorally relevant information as well. Third, all of the FC-related analyses except those involving FC fingerprints were carried out at the network-to-network level, instead of the ROI-to-ROI level, which is commonly used in FC-based behavioral prediction studies (Shen et al., 2017). The use of such network-averaged connectivity measures meant that plenty of behaviorally relevant information at the ROI level was diluted; the rsFC-behavior and rsFC-rsTC associations might have been stronger if the analyses were carried out at the ROI-to-ROI level. Nevertheless, we chose to focus on the network-to-network level such that our results would be more relatable to the rsFC literature since rsFC-related findings were often referred to and discussed at the network-to-network level. Fourth, although we included many different behavioral measures in our analyses, none of these included objectively measured cognitive abilities such as attention, executive functions, and memory. Thus, it remains unclear if rsTC can similarly mediate the relationship between rsFC and cognitive abilities. Finally, although we included subcortical regions in our FNC analyses, the functional connections originating from the 19 different subcortical regions were aggregated into a single ‘subcortical network’. As such, information from specific subcortical regions (e.g., hippocampus and amygdala), which could be useful in predicting rsTC and behavior, was not utilized.

Despite these limitations, our findings present major implications for the rsfMRI field. In most rsfMRI studies, rsTC was rarely measured or mentioned. Our findings strongly encourage future rsfMRI research to include a measure of rsTC; these measurements are quick and easy to administer. Such rsTC data can offer meaningful explanations and interpretations to bridge the rsFC-behavior gap. That is, individuals with certain behavioral traits mind-wander in a certain manner and such patterns of mind-wandering, in turn, are related to their rsFC fingerprints. Such explanations make much more sense compared to attempts to link certain patterns of rsFC to psychological processes (e.g., executive control) that were not elicited during the resting-state scan. Next, our findings in relation to vigilance and rsFC would be of major interest to rsFC fingerprint researchers. In particular, we showed that similarity in vigilance most strongly predicted similarity in rsFC fingerprints. This suggests that variations in vigilance levels could be associated with significant perturbations of one’s rsFC fingerprints, consequently making it difficult for researchers to correctly identify an individual with their rsFC fingerprints. In relation to this, it was shown that the identifiability of a subject using FC fingerprints derived from magnetoencephalography (MEG) recordings at baseline was significantly reduced when the subject was sleep deprived (hence low in vigilance) during the subsequent MEG recording (Ambrosanio et al., 2024). Beyond FC fingerprinting studies, such vigilance-related FC instability is likely to confound rsFC-behavior associations as well. If variations in vigilance levels across 1 hour can influence FNC in a fairly widespread manner across the brain, one can imagine such effects on rsFC will be even more salient if extraneous influences on vigilance, such as coffee consumption and time of the day of the rsfMRI scan, are not adequately controlled for. Thus, future rsfMRI research may want to control for these factors as much as practicable.

## Supplementary Material

Supplementary Material

## Data Availability

The R codes for the analyses and generating the figures, together with the preprocessed data analyzed in this study are available athttps://osf.io/5hkdg/. The raw MRI and behavioral data used in the current study can be accessed fromhttps://openneuro.org/datasets/ds000221/versions/1.0.0andhttps://doi.org/10.7910/DVN/VMJ6NV, respectively.
