# Supplementary material for: Thought contents during rest account for functional connectivity-behavior associations
Source: Imaging Neurosci (Camb). 2025 Jun 20;3:IMAG.a.55. doi: 10.1162/IMAG.a.55 (PMC12319998; doi:10.1162/IMAG.a.55)
Supplement: Supplementary Material [file imag.a.55_supp.pdf]

Power analysis

In relation to the first hypothesis, no previous studies have analyzed the relationship between similarity in rsFC and rsTC, as we have done. Hence, we did not have a reference effect size estimate. To this end, we made a conservative assumption that the magnitude of the relationship between similarity in rsFC and rsTC is characterized by a small (β=.02) effect size and estimated the statistical power in detecting such small effect sizes.

We ran simulation analyses to estimate this statistical power. In these analyses, we generated artificial data, with the same sample size ($N_{subjects}$=164) and total number of observations ($N_{observations}$=646) as our actual analyses, for the fixed and random effects and error by sampling from a normal distribution with a mean=0 and SD=1. The coefficient of the fixed effect is set at 0.2. Since we did not have a reasonable assumption on what the coefficient of the fixed intercept should be, we iterated the power analyses across 11 values, from 0 to 1 in increments of 0.1. For each of the iterated fixed intercept values, 1000 simulations were carried out. Statistical power was calculated as the proportion of simulations where the statistical significance of the predictor's coefficient is smaller than a preset alpha value of 0.001; we used a more conservative alpha value to approximate the effect of correcting for false discovery rate. Across the 11 iterated intercept values, the statistical power ranged from 98.7% to 99.4%

For the second set of linear mixed effect analyses addressing the second hypothesis, likewise, we did not have any previous effect estimates we can rely on, so we estimated the statistical power associated with detecting the same small effect size. We used the same procedures and parameters as above except that the artificial data was generated for 646 observations to match the number of observations in the actual analyses. Across the 11 iterated intercept values, the statistical power ranged from 87.9% to 91.1%

Finally, we estimated the statistical power of our mediation analyses in relation to the third hypothesis. This was also carried out with simulations. Unlike the previous two sets of power estimation, we had previous mediation path estimates, as reported in figure 5a of Vatansever et al[1], which we could make use of. We specified these mediation path estimates in our simulations and similarly carried out 1000 simulations. The predictor values and errors were sampled from a normal distribution with a mean=0 and SD=1. Statistical power was calculated as the proportion of simulations where the statistical significance of the bootstrapped indirect effect is smaller than a preset alpha value of 0.001. The statistical power for these mediation analyses was estimated to be 99.7%.

The R codes for running these power analyses are available at https://osf.io/5hkdg/

Table 1. All analyzed measures listed in figures 3 and 4

Abbreviated labels	Full name of measure/scale
ACS	Attention Control Scale[3]
ASR_adaptiveFunctioning_family	Adult Self Report[4]: Adaptive Functioning (Family)
ASR_adaptiveFunctioning_friends	Adult Self Report[4]: Adaptive Functioning (Friends)
ASR_aggressiveBehavior	Adult Self Report[4]: Aggressive Behavior Syndrome Profile
ASR_anxiousdepressed	Adult Self Report[4]: Anxious Depressed Syndrome Profile
ASR_attentionProblems	Adult Self Report[4]: Aggressive Behavior Syndrome Profile
ASR_criticalItems	Adult Self Report[4]: Critical Items
ASR_externalizing	Adult Self Report[4]: Externalizing Syndrome Profile
ASR_internalizing	Adult Self Report[4]: Internalizing Syndrome Profile
ASR_intrusive	Adult Self Report[4]: Intrusive Syndrome Profile
ASR_rulebreakingBehavior	Adult Self Report[4]: Rule Breaking Syndrome Profile
ASR_somaticComplaints	Adult Self Report[4]: Somatic Complaint Syndrome Profile
ASR_thoughtProblems	Adult Self Report[4]: Thought Problems Syndrome Profile
ASR_withdrawn	Adult Self Report[4]: Withdrawn Syndrome Profile
BDI	Beck Depression Inventory-II[5]
BISBAS_BAS	Behavioral Inhibition And Approach System[6]: Behavioral Inhibition System
BISBAS_BIS	Behavioral Inhibition And Approach System[6]: Behavioral Approach System
BPS	Boredom Proneness Scale[7]
ESS	Epworth Sleepiness Scale[8]
GoldMSI_Active	Goldsmiths Musical Sophistication Index[9]: Active Engagement
GoldMSI_Training	Goldsmiths Musical Sophistication Index[9]: Musical Training
HADSA	Hospital Anxiety And Depression Scale[10]: Anxiety
HADSD	Hospital Anxiety And Depression Scale[10]: Depression
IAT	Internet Addiction Test[11]
IMIS_Help	Involuntary Musical Imagery Scale[12]: Help
IMIS_Movement	Involuntary Musical Imagery Scale[12]: Movement
IMIS_NegVal	Involuntary Musical Imagery Scale[12]: Negative Valence
IMIS_PersRef	Involuntary Musical Imagery Scale[12]: Personal Reflections
MMI_score	Multimedia Multitasking Index[13]
PSSI_AB	Personality Style And Disorder Inventory[14]: Loyal/Dependent
PSSI_AS	Personality Style And Disorder Inventory[14]: Autonomous/Antisocial
PSSI_BL	Personality Style And Disorder Inventory[14]: Spontaneous/Borderline
PSSI_DP	Personality Style And Disorder Inventory[14]: Quiet/Depressive
PSSI_HI	Personality Style And Disorder Inventory[14]: Endearing/Histrionic
PSSI_NA	Personality Style And Disorder Inventory[14]: Ambitious/Narcissistic
PSSI_NT	Personality Style And Disorder Inventory[14]: Critical/Negativistic
PSSI_PN	Personality Style And Disorder Inventory[14]: Self-Willed/Paranoid
PSSI_RH	Personality Style And Disorder Inventory[14]: Optimistic/Rhapsodic
PSSI_SL	Personality Style And Disorder Inventory[14]: Helpful/Selfless
PSSI_ST	Personality Style And Disorder Inventory[14]: Suspecting/Schizotypic
PSSI_SU	Personality Style And Disorder Inventory[14]: Self-Critical/Insecure
PSSI_SZ	Personality Style And Disorder Inventory[14]: Reserved/Schizoid

PSSI_ZW	Personality Style And Disorder Inventory[14]: Careful/Compulsive
SDMW_delib	Spontaneous And Deliberate Mind Wandering[15]: Deliberate Mind Wandering
SDMW_spont	Spontaneous And Deliberate Mind Wandering[3]: Spontaneous Mind Wandering
SCS_SelfCtrl	Brief Self-Control Scale[16]
SD3_Mach	Short Dark Triad[17]: Machiavellianism
SD3_Narc	Short Dark Triad[17]: Narcissism
SD3_Psycho	Short Dark Triad[17]: Psychopathy
SDS	Social Desirability Scale-17[17]
SE_SelfEst	Self-Esteem Scale[18]
STAXI_anger_control	State-Trait Anger Expression Inventory[19]: Anger Control
STAXI_anger_inward	State-Trait Anger Expression Inventory[19]: Anger Inward
STAXI_anger_outward	State-Trait Anger Expression Inventory[19]: Anger Outward
STAXI_anger_trait	State-Trait Anger Expression Inventory[19]: Anger Trait
TPS_D	Tuckman Procrastination Scale[20]
UPPS_NegUrg	Impulsive Behavior Scale[21]: Negative Urgency
UPPS_Persev	Impulsive Behavior Scale[21]: Lack Of Perseverance
UPPS_PosUrg	Impulsive Behavior Scale[21]: Positive Urgency
UPPS_Premed	Impulsive Behavior Scale[21]: Lack Of Premeditation
UPPS_SS	Impulsive Behavior Scale[21]: Sensation Seeking
VIS_condensed	Varieties Of Inner Speech Questionnaire[22]: Condensed Inner Speech
VIS_dialog	Varieties Of Inner Speech Questionnaire[22]: Dialogic Inner Speech
VIS_eval	Varieties Of Inner Speech Questionnaire[22]: The Voice Of Others In The Inner Speech
VIS_other	Varieties Of Inner Speech Questionnaire[22]: Condensed Inner Speech

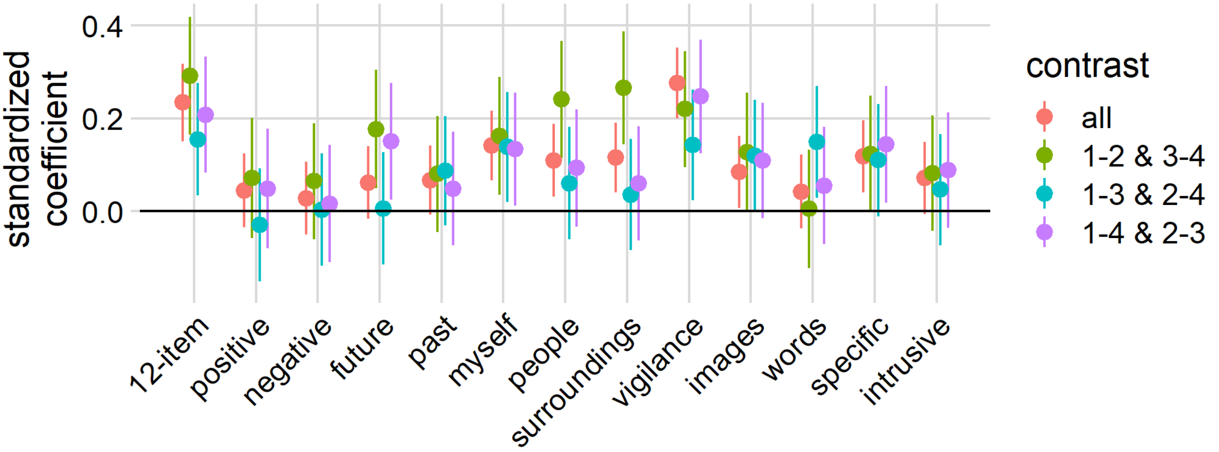

Figure S1. Standardized coefficients and their respective 95% confidence intervals when the MAD_{SNYCQ} and absolute differences in item scores were used to predict the MAD_{FC} in linear mixed effects models. These coefficients are statistically significant if their 95% confidence intervals (uncorrected for multiple comparisons) do not intersect with the y=0 line. The red data points represent the original results as shown in figure 3a in the main text. The green, blue and purple data points represent the results when non-overlapping contrasts were analyzed.

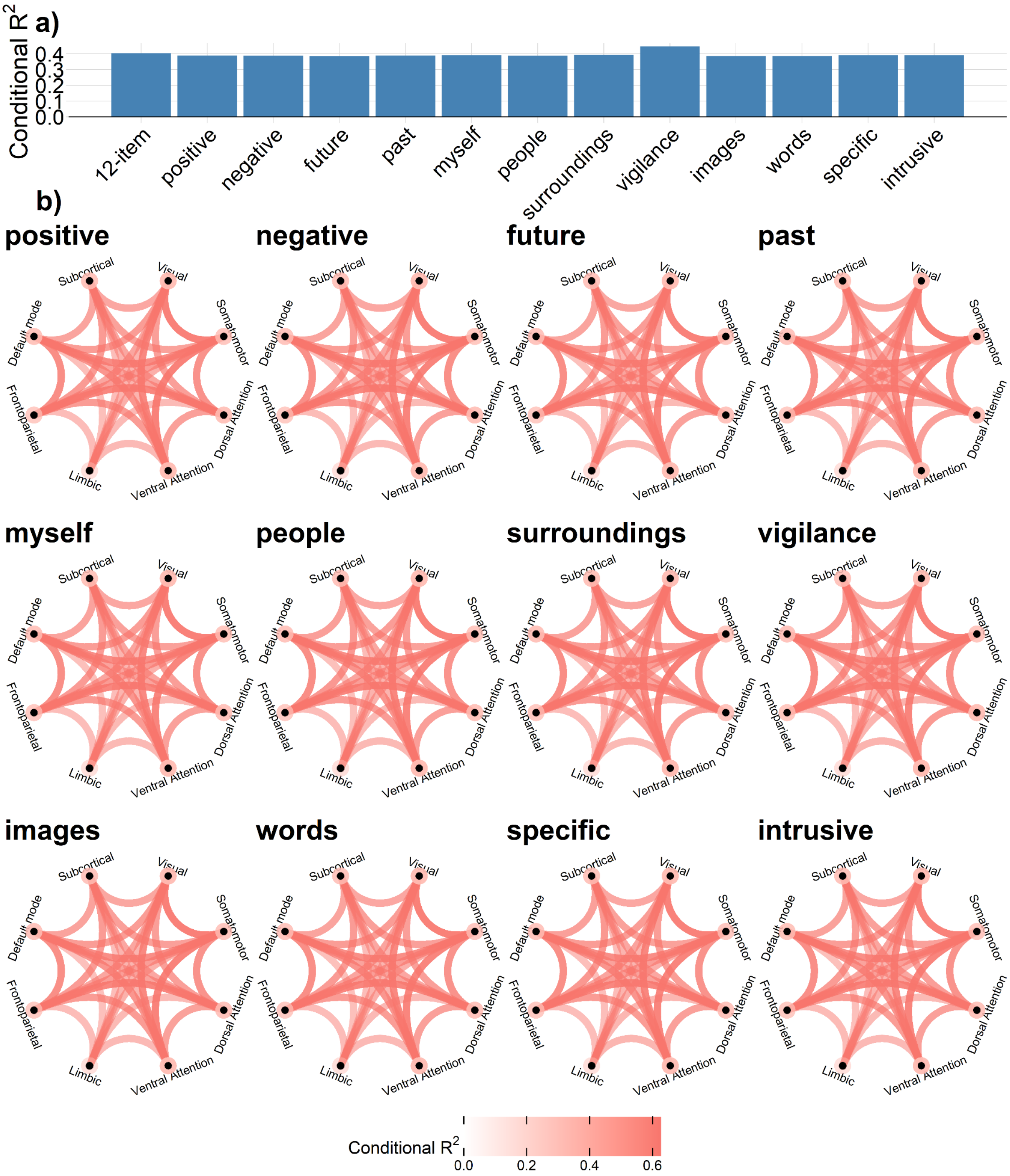

Figure S2. a) conditional R^2 when the MADSNYCQ and absolute differences in item scores were used to predict the MADFC in linear mixed effects models. b) Connectograms for each of the SNYCQ items illustrating the conditional R^2 of the network-to-network connections. The rings around

the nodes represent within-network connections.

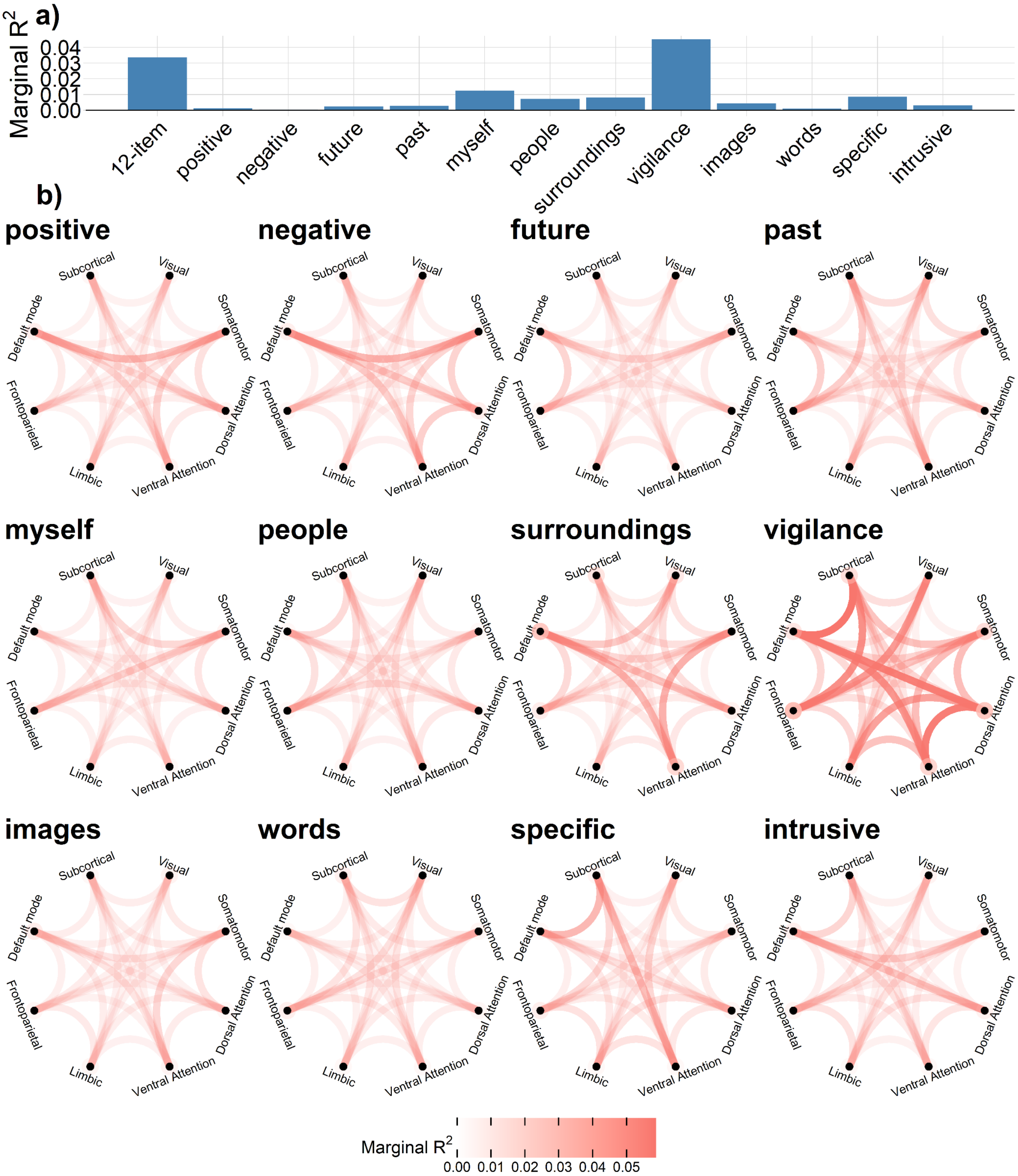

Figure S3. a) Marginal R^2 when the MADSNYCQ and absolute differences in item scores were used to predict the MADFC in linear mixed effects models. b) Connectograms for each of the SNYCQ items illustrating the marginal R^2 of the network-to-network connections. The rings around the nodes represent within-network connections.

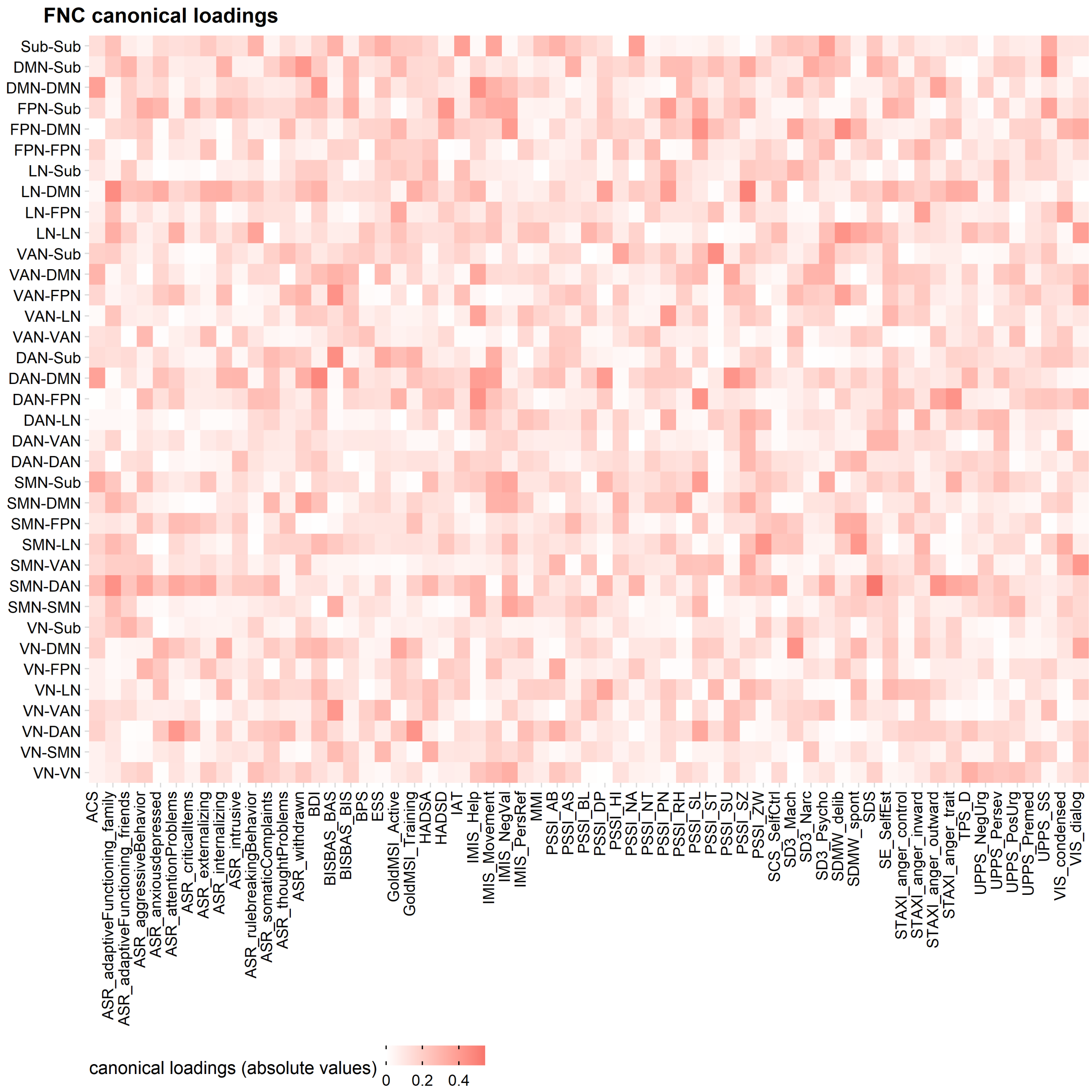

Figure S4. canonical loadings (absolute values) of FNC edges. VN=Visual network; SMN=Somatomotor Network; DAN=Dorsal Attention Network; VAN=Ventral Attention Network; LN=Limbic Network; FPN=Frontoparietal Network; DMN=Default Mode Network; Sub=Subcortical regions

Supplementary analyses on participants aged ≤ 40

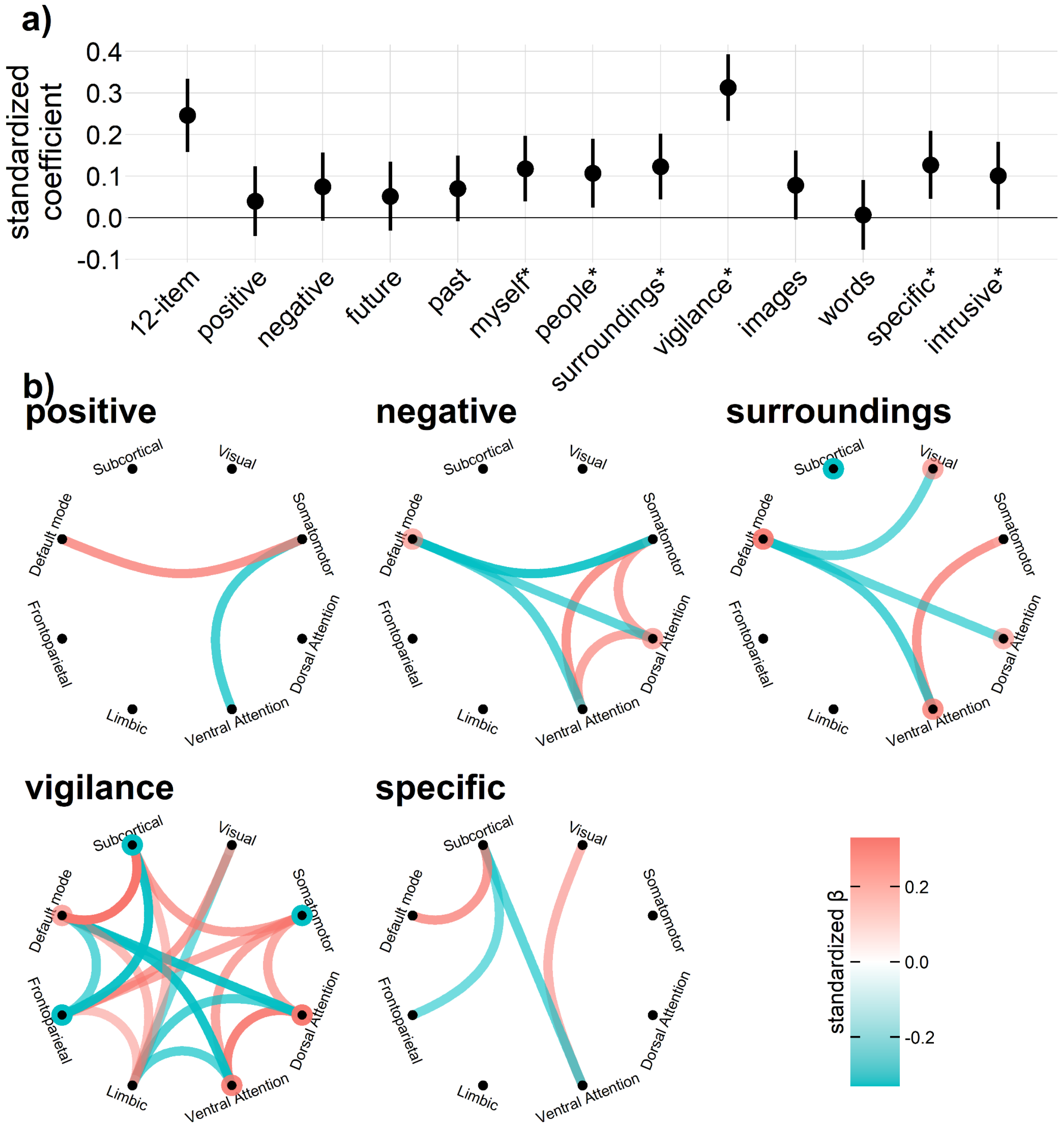

Figure S5. a) Standardized coefficients and their respective 95% confidence intervals when the MAD_{SNYCQ} and absolute differences in item scores were used to predict the MAD_{FC} in linear mixed effects models. These coefficients are statistically significant if their 95% confidence intervals (uncorrected for multiple comparisons) do not intersect with the y=0 line.

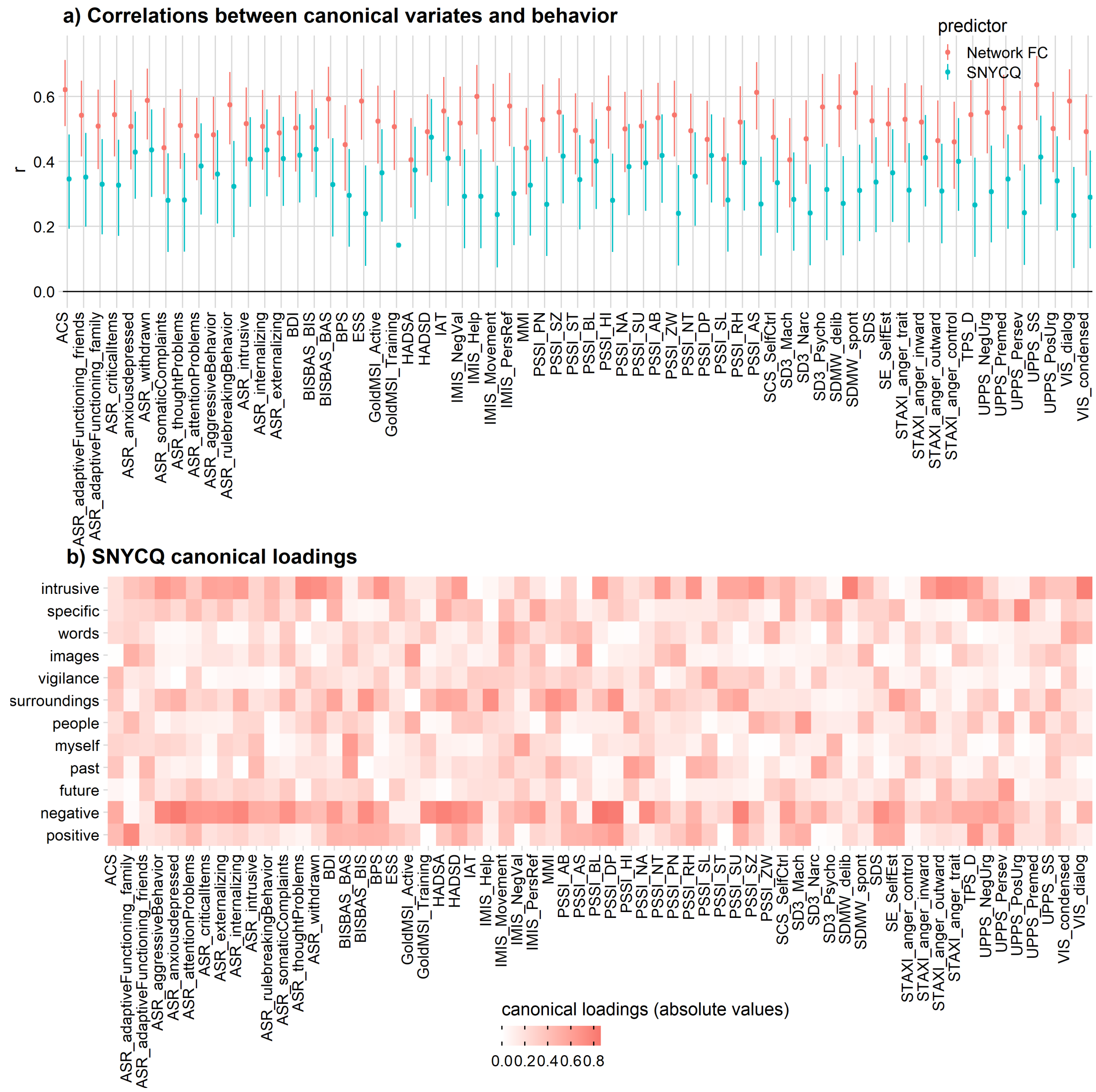

Figure S6. a) correlations between the CV_{FNC} and CV_{SNYCQ} and behavioral phenotypes. The error bars represent the coefficients' 95% confidence intervals. These coefficients are statistically significant if their 95% confidence intervals do not intersect the y=0 line. b) canonical loadings (absolute values) of SNYCQ items.

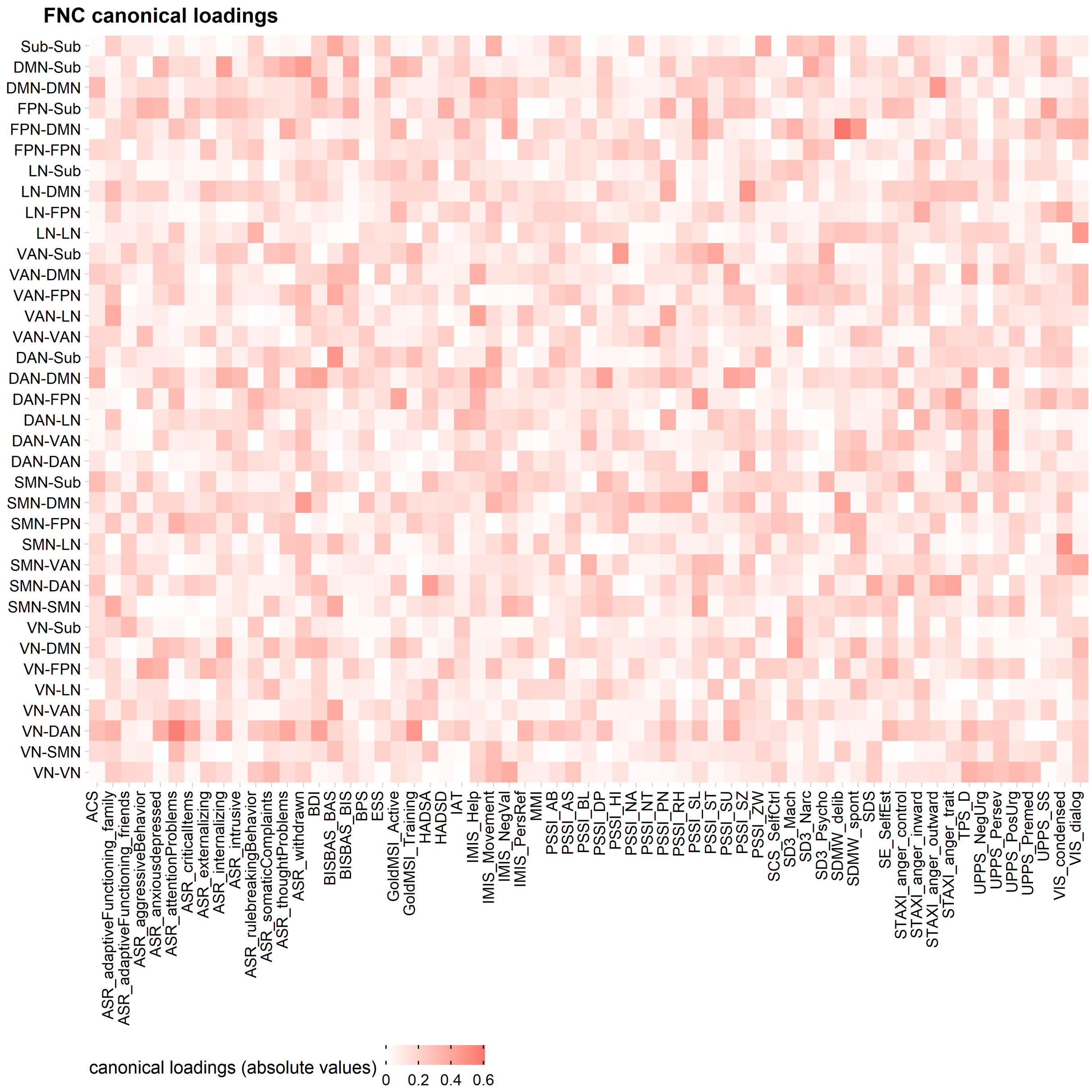

Figure S7. canonical loadings (absolute values) of FNC edges. VN=Visual network; SMN=Somatomotor Network; DAN=Dorsal Attention Network; VAN=Ventral Attention Network; LN=Limbic Network; FPN=Frontoparietal Network; DMN=Default Mode Network; Sub=Subcortical regions

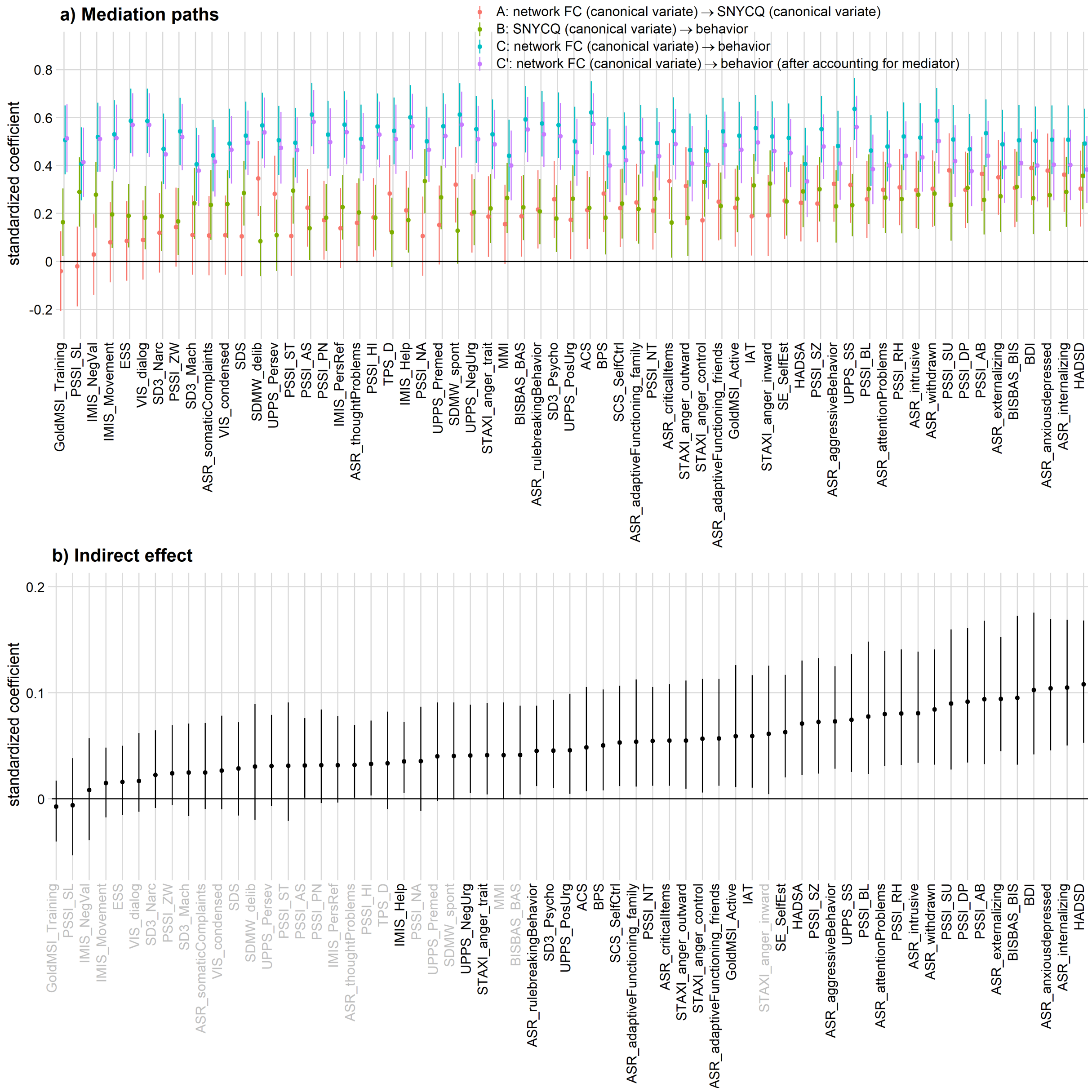

Figure S8. a) Standardized coefficients of the three mediation paths (i.e., A, B, and C) and their confidence intervals. Path A represents the coefficient of CV_{FNC} when CV_{SNYCQ} is regressed on CV_{FNC}. Path B represents the coefficient of CV_{SNYCQ} when the behavioral outcome is regressed on CV_{FNC} and CV_{SNYCQ}. Path C represents the coefficient of CV_{FNC} when the behavioral outcome is regressed on CV_{FNC}. Path C' represents the coefficient of CV_{FNC} when the behavioral outcome is regressed on CV_{FNC} and CV_{SNYCQ}. b) the indirect effect of CV_{SNYCQ} in mediating the relationship between CV_{FNC} and behavioral outcome and its confidence intervals. The error bars in both panels represent the coefficients' 95% confidence intervals. These coefficients are statistically significant (uncorrected for multiple comparisons) if their 95% confidence intervals do not intersect the y=0 line. The labels that are colored in gray correspond to indirect effects that were not statistically significant after correcting for false discovery rate. The full names of these measures and their references are provided in the supplementary materials (see table S1). For both panels, the behavioral phenotypes are arranged along the x-axis in order of increasing indirect effects.

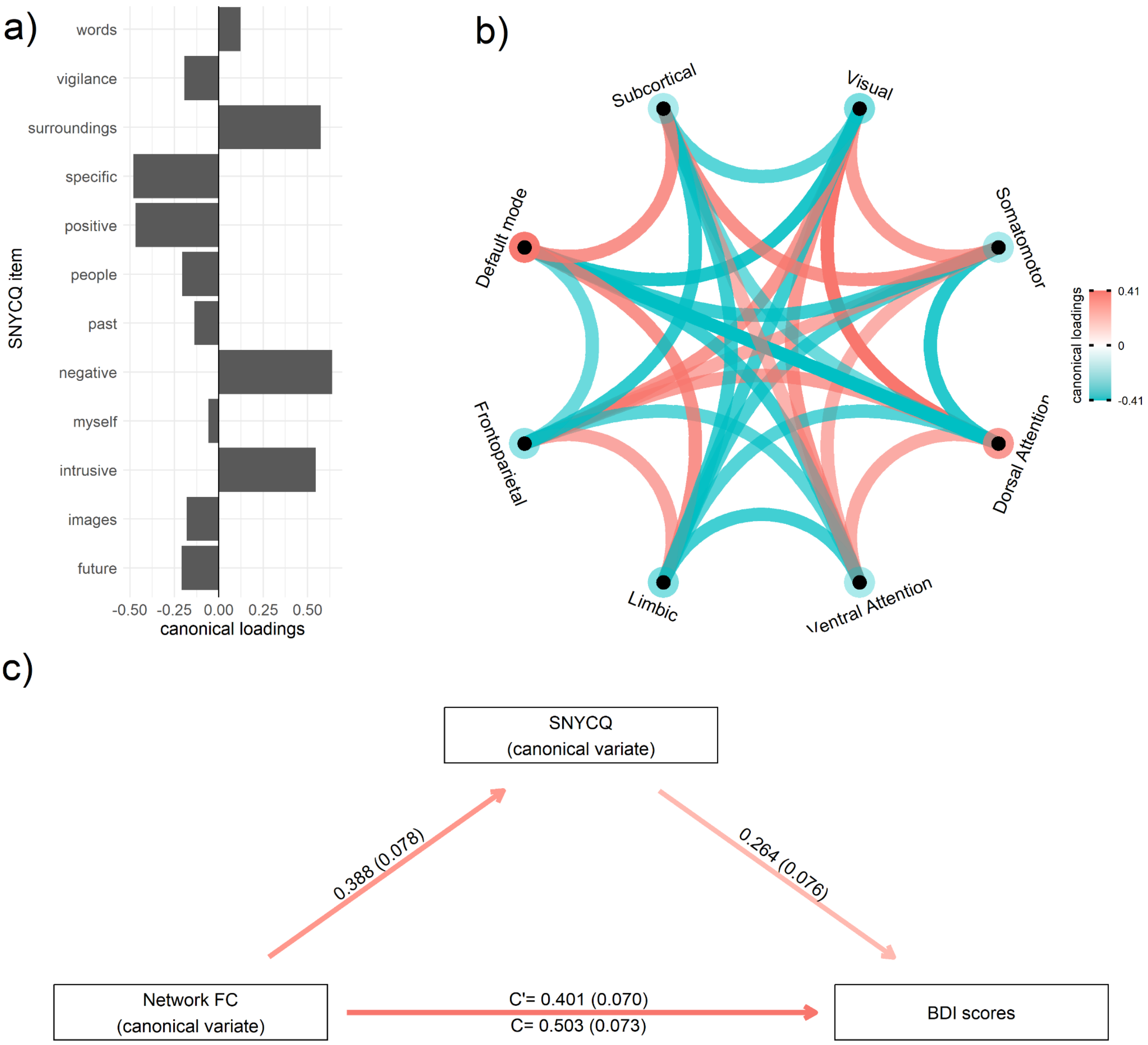

Figure S9. Example of the analyses involving the Beck Depression Inventory scores. a) SNYCQ item loadings from the canonical correlation analysis and b) network FC. c) mediation path diagram. All reported coefficients in the mediation path diagram are standardized and statistically significant ($p<.001$). The standard errors of the coefficients are provided in parentheses.

References

1. Vatansever, D., Karapanagiotidis, T., Margulies, D. S., Jefferies, E. & Smallwood, J. Distinct patterns of thought mediate the link between brain functional connectomes and well-being. *Network Neuroscience* **4**, 637–657 (2020).
2. Stoffers, D. *et al.* Resting-State fMRI Functional Connectivity Is Associated with Sleepiness, Imagery, and Discontinuity of Mind. *PLOS ONE* **10**, e0142014 (2015).
3. Derryberry, D. & Reed, M. A. Anxiety-related attentional biases and their regulation by attentional control. *Journal of Abnormal Psychology* **111**, 225–236 (2002).
4. Achenbach, T. M. & Rescorla, L. *Manual for the ASEBA Adult Forms & Profiles: For Ages 18-59 : Adult Self-Report and Adult Behavior Checklist*. (ASEBA, 2003).
5. Beck, A. T., Steer, R. A. & Brown, G. Beck Depression Inventory–II. https://doi.org/10.1037/t00742-000 (2011).
6. Carver, C. S. & White, T. L. Behavioral inhibition, behavioral activation, and affective responses to impending reward and punishment: The BIS/BAS Scales. *Journal of Personality and Social Psychology* **67**, 319–333 (1994).
7. Farmer, R. & Sundberg, N. D. Boredom Proneness--The Development and Correlates of a New Scale. *Journal of Personality Assessment* **50**, 4–17 (1986).
8. Bloch, K. E., Schoch, O. D., Zhang, J. N. & Russi, E. W. German Version of the Epworth Sleepiness Scale. *Respiration* **66**, 440–447 (1999).
9. Müllensiefen, D., Gingras, B., Musil, J. & Stewart, L. The Musicality of Non-Musicians: An Index for Assessing Musical Sophistication in the General Population. *PLOS ONE* **9**, e89642 (2014).
10. Zigmond, A. S. & Snaith, R. P. The Hospital Anxiety and Depression Scale. *Acta Psychiatrica Scandinavica* **67**, 361–370 (1983).
11. YOUNG, K. S. Internet Addiction: The Emergence of a New Clinical Disorder. *CyberPsychology & Behavior* **1**, 237–244 (1998).
12. Floridou, G. A., Williamson, V. J., Stewart, L. & Müllensiefen, D. The Involuntary Musical Imagery Scale (IMIS). *Psychomusicology: Music, Mind, and Brain* **25**, 28–36 (2015).
13. Ophir, E., Nass, C. & Wagner, A. D. Cognitive control in media multitaskers. *Proceedings of the National Academy of Sciences* **106**, 15583–15587 (2009).
14. Kuhl, J. & Kazén, M. *Persönlichkeits-Stil- Und Störungs-Inventar: PSSI ; Manual*. (Hogrefe, 2009).

15. Carriere, J. S. A., Seli, P. & Smilek, D. Wandering in both mind and body: Individual differences in mind wandering and inattention predict fidgeting. *Canadian Journal of Experimental Psychology / Revue canadienne de psychologie expérimentale* **67**, 19–31 (2013).
16. Tangney, J. P., Baumeister, R. F. & Boone, A. L. High Self-Control Predicts Good Adjustment, Less Pathology, Better Grades, and Interpersonal Success. *Journal of Personality* **72**, 271–324 (2004).
17. Jones, D. N. & Paulhus, D. L. Introducing the Short Dark Triad (SD3): A Brief Measure of Dark Personality Traits. *Assessment* **21**, 28–41 (2014).
18. O'Malley, P. M. & Bachman, J. G. Self-esteem and education: Sex and cohort comparisons among high school seniors. *Journal of Personality and Social Psychology* **37**, 1153–1159 (1979).
19. Spielberger, C. D. State-trait anger expression inventory research edition. *Professional manual. Odessa, FL: Psychological Assessment Resources* (1988).
20. Tuckman, B. W. The Development and Concurrent Validity of the Procrastination Scale. *Educational and Psychological Measurement* **51**, 473–480 (1991).
21. Lynam, D. R., Smith, G. T., Whiteside, S. P. & Cyders, M. A. The UPPS-P: Assessing five personality pathways to impulsive behavior. *West Lafayette, IN: Purdue University* **10**, (2006).
22. McCarthy-Jones, S. & Fernyhough, C. The varieties of inner speech: Links between quality of inner speech and psychopathological variables in a sample of young adults. *Consciousness and Cognition* **20**, 1586–1593 (2011).
